# Control study of *Musca domestica* (Diptera, Muscidae) in Misan Province

**DOI:** 10.12688/f1000research.132636.1

**Published:** 2023-05-02

**Authors:** Rasha Alsaad

**Affiliations:** 1 Microbiology Department, Faculty of Medicine, Misan University, Misan, 62001, Iraq

**Keywords:** Musca domestica, Housefly, Container Index, House Index, larvae

## Abstract

**Background:** Houseflies are the most common type of Diptera, specifically Muscidae, worldwide, representing more than 90% of all species. This family has over 170 genera and 4200 species, but a few are of medical significance. This study aimed to estimate and assessing the measures to control and prevent grow-up inside houses and flying of the housefly ( *Musca domestica Linnaeus*, 1758) in Misan.

**Methods:** The study occurred over 12 months, from December 2020 to December 2021. Using plastic containers, *Musca* flies were collected from all potential breeding sites in the study region (inside and around houses). Sticky oil paper and traps were used to collect the insects. The collected insects were transferred to sealed plastic containers and then to the laboratory of the Department of Microbiology.

**Results:** Out of 200 randomly selected houses, 150 (75%) contained insects. Light traps and sticky oil papers were the most effective control measures, with 26.7% and 25.9% of the *Musca* collected from these methods, respectively. The ratio of male (233) to female (456) *Musca* was 1:2, with a significant difference between the frequencies (P<0.05). A large population of houseflies was collected during the hot season (501, 72.7%), whereas fewer *Musca* were collected during the cold months (188, 27.3%), with a strongly significant difference (P<0.05). The percentage of HI was 54.4%, the CI was 21.9%, and the BI was 79.9%. The overall larval densities (LD) were at a medium level.

**Conclusions:** Misan has a high density of *Musca* houseflies, with females being more prominent than males. Hot climate, humid sites, and dirty places are responsible for the breeding of houseflies. The overall larval density was medium. Therefore, the risk of transmitting infectious diseases by houseflies is high within the boundaries of Misan province, and effective control parameters should include measures like light traps and sticky oil.

## Introduction

*Musca* (Order Diptera, family Muscidae) is a major concern for human health because they are vectors of many tropical and subtropical diseases. ^1^^,^^2^ Houseflies are abundant in regions like dirty places; they prefer warm environments and occur in moist regions during the daytime. ^3^ They frequently transit around dirty places, animals, and food sources, defecating during feeding, making them ideal disease vectors for spreading microorganisms. Several studies have reported that these vectors transmit several communicable illnesses via pathogens collecting on their body parts, such as female laying eggs on decomposed materials. ^4^^,^^5^ Houseflies can transmit leprosy, anthrax, tuberculosis (TB), dysentery, typhoid, diphtheria, and gastrointestinal parasites. Additionally, they play a role as mechanical vectors or intermediate hosts for nematodes and cestodes. ^6^

In developing countries, these flies can cause the spreading of gastroenteritis and trachoma among children because they have thinner skin, play outside the home and mostly don’t cover their whole body by clothes, and they can also transmit nosocomial infections in hospital environments. ^7^^–^^13^ Mullen and Durden ^14^ and Sales *et al.* ^15^ identified different parasites and worm eggs in fly feces, including *Diphyllobothrium* sp., *Trichuris trichiura*, *Hymenolepis* sp., *Strongyloides stercoralis*, *Ascaris lumbricoides*, *Enterobius vermicularis*, *Toxocara cani*, *Giardia* sp., *Taenia* sp., *Trichomonas* sp., *Entamoeba histolytica*, and fungi.

Houseflies can be controlled through physical and chemical means. Vector control is the most common method for protecting communities against vector-borne illnesses. ^16^^–^^18^ Traditionally, control processes have focused on killing insects by using different insecticides. Environmental treatment involves removing breeding sites through microbiological ovicides, chemical larvicides and pupacides in regions where endemic-borne diseases occur. ^17^ *Musca* breeds can live in various habitats, including freshwater habitats, water in mangrove forests, septic tanks, domestic waste, desert coolers, indoor and outdoor environments with stagnant water conditions as humidity, clear roots of aquatic plants, and damp places. ^18^ It takes seven to 10 days for *Musca* to complete its life cycle under good conditions as median temperature and moderate humidity and not rainy weather, while it can take up to two months under poor conditions like hotter temperatures, heavy rain and colder weather. In temperate locations, twelve generations may occur during one year, whereas in the tropics and subtropics, it may take more than 20 generations. ^19^ Evaluating larval habitats in terms of species composition and resources helps to understand the ecology, control measures, and proper identification of different species, as well as monitoring density, which is important for controlling houseflies. ^20^^–^^23^

This study aimed to estimate and assess the measures used to control the housefly ( *Musca domestica Linnaeus*, 1758) in Misan.

## Methods

### Ethics approval

The approval was granted by the University of Misan, Faculty of Medicine Committee Board (ID No. 103/Oct 2020).

• Ethical committee approved the selection of private houses for the study: Faculty of Medicine, University of Misan
• The approval number: 103

### Study regions

Misan province comprises six sub-districts, including Al-Amarah, Al-Kehlaa, Ali Al-Gharbi, Al-Majar Alkabeer, Qal’at Saleh, and Al-Maymouna. The study occurred over 12 months, from December 2020 to December 2021.

### Insect collection

**Figure 1.**
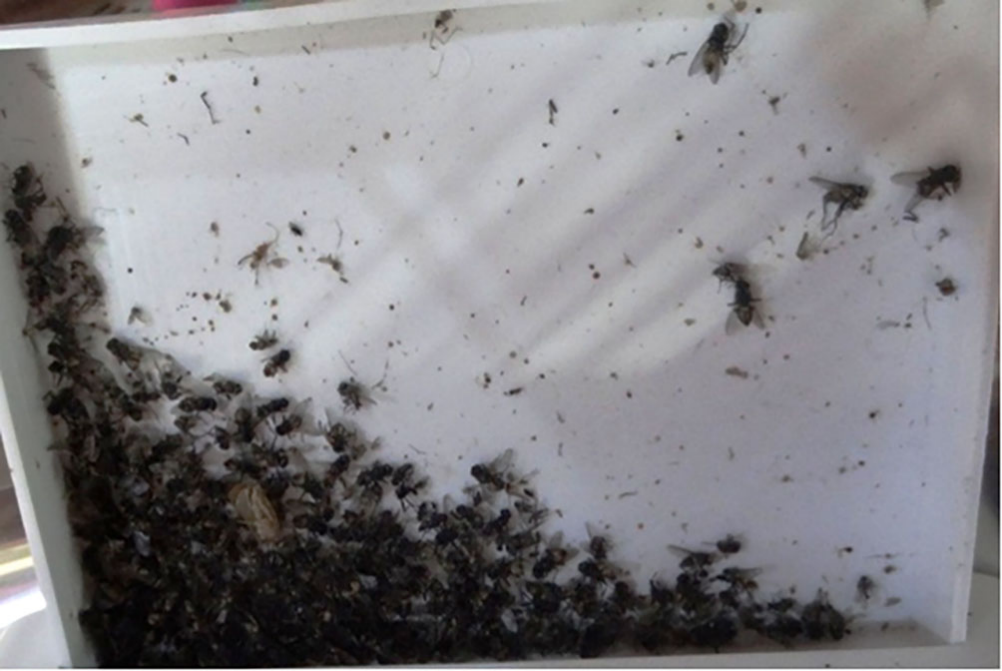
Container for collecting insects.

Using plastic containers *, Musca* flies were collected from all potential breeding sites in the study region (inside and around houses). Sticky oil paper (insect glue snares) (FLYING, China, Cat. No. 15-B) and traps containing light (Moth UV light trap, Japan, Cat. No. DSCF199055) were used to collect the insects by placing papers and traps randomly indoor and outdoor the houses and in the collecting habitats. They were then placed in Petri dishes (ATACO, China, Cat. No. 34809) which were filled with distilled water (BDH, UK, Cat. No. 45550W) according to Alsaad and Kawan ^24^ and kept till the time of examination and identification ( Figure 1).

The collected insects were transferred to sealed plastic containers and then to the laboratory of the Department of Microbiology. The houseflies were identified based on their shapes, morphologies, and sizes using single concave microscope slides which are helpful in fixing insect (AmScope, US, Cat. No. 660LOO) and anatomical microscope (Olympus, Japan, Cat. No. 2033789). The adult male housefly has reddish eyes positioned closely to each other, and spongious mouthparts. The male is 5–8 mm in length and has four dark stripes on a dull gray thorax, on the dorsal side and pronounced upward bends in the fourth longitudinal wing vein. The basal portions of the abdomen are yellowish, especially on both sides’ alignment. Typically, males show a greater laterally yellowish color than females. Dark longitudinal bands run along the median dorsal region of the anterior portion of abdominal segments. Adult female houseflies’ eyes are more widely separated than males’. In female houseflies, the bends in the fourth longitudinal wing veins are distinctly more upward than in males. The female is 3–8 mm in length, and have a lightly golden checkered abdomen, more so than males, according to Yeates *et al.* ^25^ and Geden *et al*. ^26^

### Study equipment

The tools used in the study included pipettes (Quawell, USA, Cat. No. R2033), plastic bottles (EASTMED, China, Cat. No. T75400), plastic bags (ATACO, China, Cat. No. 38662), specimen stoppers (ATACO, China, Cat. No. 56988), pens (ATACO, China, Cat. No. 10076), and sticky oil paper (Verlge, Germany, Cat. No. 38662).

### Larval densities

Three insect indices, including the Breteau Index (BI), Container Index (CI), and House Index (HI), were measured. In BI, If the percentage of >20%, this represents a high risk of transmission while If it <20% is considered low risk for BI. In CI, if percentage >50%, this represents a high risk of disease transmission, whereas if it <50%, this reflects a low risk for CI. In HI, if percentage ≥5%, this represents a high risk of transmission while that <5% consider low risk for HI. ^27^ The density of the larval index description is a combination of HI, CI, and BI and is rated on a scale of 1–9, as shown in Table 1, according to the Queensland Government. ^28^ The indices scoring is subdivided into three groups: LD = 1 for low, LD = 2–5 for medium, and LD = 6–9 for high ( Table 1).

**Table 1.** Larva densities, indices and degrees. ^27^

LD	HI	CI	BI	Degree
1-2	1 – 3	1 – 2	1 – 4	Low
3-5	4 – 37	3 – 20	5 – 49	Medium
6-9	38 – />77	21 – />41	50 – />200	High

HI = house index, CI = container index, BI = breteau index, LD = Larval densities.

### Statistical analysis

All findings were analyzed using SPSS Statistics version 22 (IBM Corporation, New York, US). Categorical data were presented as frequencies and percentages. The chi-square (χ ^2^) test was used to describe the association between categorical variables, with a P-value of <0.05 considered statistically significant.

## Results

To select the houses, the author visited every city of Misan province by car as well as each town and village of that city. When reaching the place, the author went through the ethics process from their Institution. Additionally, I used my identity card of College of Medicine to take permission for placed papers and traps in collecting regions) Out of 200 randomly selected houses, 150 (75%) contained insects, while 50 (25%) did not have any larvae. Houses were the location of samples collection. The housefly collected in this study were collected from these houses by the homeowners using traps and sticky papers supplied by the researcher. I distributed papers and traps to the household owner for free, and collected insects at every visit. The householder gave me consent to collect insects from papers and traps that I bought and gave them freely. Houses were selected for being nearby large water sources and farms or many trees, or regions of good conditions for grow-up of houseflies like moderate humidity and medium temperatures. The distribution of *Musca* in their habitats was as follows: indoors (48, 6.9%), outdoors (115, 16.8%), plastic cups (69, 10.0%), traps (184, 26.7%), oil papers (179, 25.9%), and sewage water (94, 13.7%) with a high statistical significance (P = 0.001). Light traps and sticky oil papers were the most effective control measures, with 26.7% and 25.9% of the *Musca* collected using these methods, respectively ( Table 2).

**Table 2.** Distribution of *Musca* in habitats.

Habitats	Number of houses	%	Number of *Musca*	%
Cups	15	7.5	69	10.0
Indoor	10	5	48	6.9
Outdoor	25	12.5	115	16.8
Light trap	40	21	184	26.7
Sticky oil	39	19	179	25.9
Sewage water	20	10	94	13.7
No insect	50	25	-	-
Total	200	100.0	689	100.0
	*X* ^2^ = 6.28, df = 4, P = 0.022		*X* ^2^ = 38.28, df = 6, P = 0.001	

**Figure 2.**
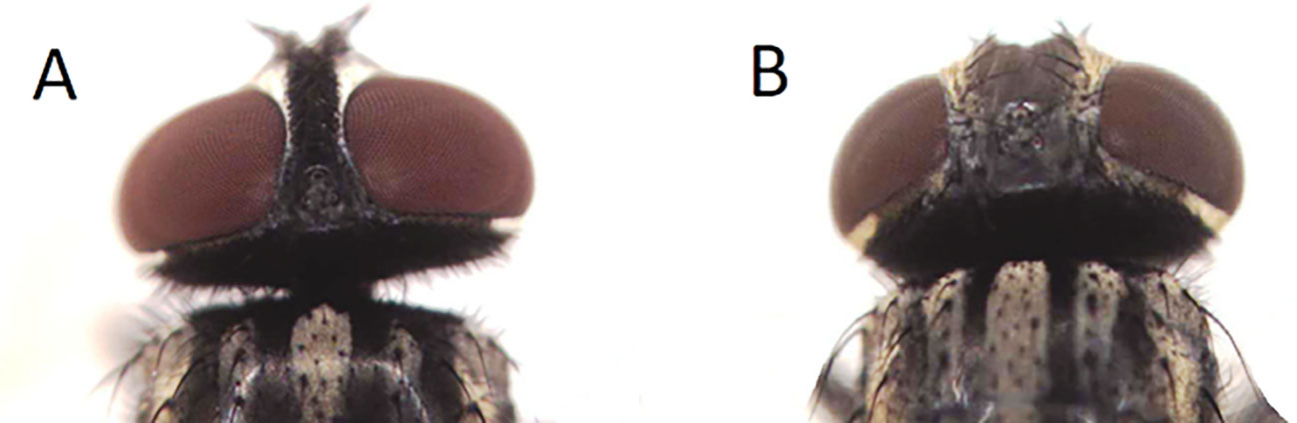
Head of male (A) and female (B) *Musca domestica* (housefly).

In this study, the ratio of male (233) to female (456) *Musca* was 1:2, with a significant difference between the frequencies (P < 0.05) ( Table 3 and Figure 2).

**Table 3.** *Musca* distribution according to sex.

Sex	Number	%
Male	233	33.8
Female	456	66.2
Total	689	100.0
*X* ^2^ = 12.14, df = 2, P < 0.05		

The study period was divided into two parts of the year: hot months (March, April, May, June, July, August, September and October) and cold months (November, December, January and February) ( Table 4). A large population of houseflies was collected during the hot season (501, 72.7%), whereas fewer *Musca* were collected during the cold months (188, 27.3%), with a strongly significant difference (P < 0.05).

**Table 4.** *Musca domestica* larvae distribution in relation to weather.

Weather	No.	%
Hot months	501	72.7
Cold months	188	27.3
Total	689	
*X* ^2^ = 10.15, df = 3, P < 0.05		

The percentage of HI was 54.4%, the CI was 21.9%, and the BI was 79.9% ( Table 5). The overall LD was at a medium level.

**Table 5.** Distribution of *Musca* according to indices.

Index	Larval densities (LD) (No.) [%]	Degree
House Index (HI)	5 (375) [54.4]	Medium
Container Index (CI)	2 (151) [21.9]	Low
Breateau Index (BI)	8 (551) [79.9]	High

## Discussion

The presence of houseflies in different habitats revealed their ability to survive in a particular environment and female oviposition preference in that habitat. Depending on the tolerance range of *Musca* spp., changes in the physio-chemical and biotic-biology features of the habitats may make other environments favorable or unfavorable to successful breeding. ^29^ According to Zulkarnaini and Dameria ^30^ and Madewell *et al.,* ^31^ when the HI, CI, and BI are greater than 50%, it indicates a high risk of illness spreading, which is very high in that population. This requires public health professionals and government to use multiple tools to control vectors such as houseflies.

Improving environmental sanitation is fundamental for achieving long-term control of houseflies. Sanitation is the mainstay for controlling houseflies in and around farms or homes. Environmental control involves cleaning garbage areas to reduce odors and prevent housefly breeding. ^32^ Insecticides, natural biological suppression of houseflies, proper management of poultry manure, flytraps, ultraviolet light traps, space sprays containing synergized pyrethrins, baits (excellent selective adulticides), Z-9-triclosan, larvicides, and Neporex are effective in controlling houseflies. ^33^^,^^34^

The breeding of flies is closely correlated with the food source where flies forage and breed. In settlements, residents throw garbage daily, attracting flies and contributing to their breeding. The density of flies is closely related to the source of infectious diseases. According to Prabowo, ^35^ *M. domestica* is found in very high densities in landfills, markets, and kitchens due to the large quantities of food processed in these areas. Arroyo ^36^ reported that they are found in many chicken farms, garbage areas, and in animal and human feces.

In this study, only *M. domestica* was collected in each region because it is commonly found in almost all places, especially food waste and dirt from human activities. ^37^ Additionally, the lifecycle of *M. domestica* requires ingesting a large amount of food, making it closely related to humans themselves. ^38^

In this study, houses were randomly selected, and 150 (75%) contained insects, while 50 (25%) had no larvae. This is similar to reports from Adenusi and Adewoga, ^39^ who mentioned that epidemiological investigations found houseflies were carriers of intestinal parasites in dirty places and garbage. Azrul ^40^ and Sigit *et al.* ^41^ documented that the flying distance of houseflies can reach between 200 to 2 km. However, the flying distance from population-dense areas is not more than 500 m, and they do not fly continuously as they often stop to forage in the garbage. Ginanjar ^42^ stated that houseflies are found in high numbers in dwellings with human activities.

In this study, the male-to-female ratio (M:F) was 1:2, and a large population of houseflies was collected during the hot season. HI, CI, and BI percentages were 54.4%, 21.9%, and 79.9%, respectively. Similar findings have been reported by several studies. ^43^^–^^50^ Many authors have stated that *M. domestica* has the highest population among flies and is widely found in residential environments, food sources, dirty cooling places, and landfills near human activities. ^41^^,^^51^^,^^52^ *Musca*’s larval life history parameters are affected by the prey quantities, types of foods, and densities of rearing of housefly. ^46^

## Conclusions

Misan province has a high density of houseflies, with females being more prominent than males. Hot climates, humid sites, and dirty places are responsible for the breeding of houseflies. The overall larval density was at a medium level. Therefore, the risk of transmission infectious diseases by houseflies is high within the boundaries of Misan province, and effective control measures such as light traps and sticky oil should be implemented.

## Data Availability

Zenodo: *Musca* Housefly distribution, https://doi.org/10.5281/zenodo.7738706. ^53^ This project contains the following underlying data:
- Musca Alsaad.xlsx (Musca domistica sex, habitats, distribution, and indices) - 1.jpg (Container for insect collection) - 2.jpg (Sticky oil paper) - 3.jpg (Sticky oil paper) - 4.jpg (Light trap) - 5.jpg (Light trap) Data are available under the terms of the Creative Commons Attribution 4.0 International license (CC-BY 4.0).
