# Supplementary material for: Control study of Musca domestica (Diptera, Muscidae) in Misan Province
Source: F1000Res. 2023 Nov 15;12:459. Originally published 2023 May 2. [Version 2] doi: 10.12688/f1000research.132636.2 (PMC10721963; doi:10.12688/f1000research.132636.2)
Supplement: Peer review history

# Reviewer report: Reviewer response for version 2

**DOI:** 10.5256/f1000research.158405.r228191

**Published:** 2024-06-14

**Authors:** Namariq Al-Saadi

**Affiliations:** 1 University of Misan, Amarah, Iraq

In detail, it is a new era in Iraq that needs to be covered. Also, the abstract and introduction are very well written. The figures and tables look nice and display the main idea for the paper. Finally, the discussion and conclusion are carefully done.

Is the work clearly and accurately presented and does it cite the current literature?

Yes

If applicable, is the statistical analysis and its interpretation appropriate?

Yes

Are all the source data underlying the results available to ensure full reproducibility?

Yes

Is the study design appropriate and is the work technically sound?

Yes

Are the conclusions drawn adequately supported by the results?

Yes

Are sufficient details of methods and analysis provided to allow replication by others?

Yes

Reviewer Expertise:

Molecular biology and genetics. General biology

I confirm that I have read this submission and believe that I have an appropriate level of expertise to confirm that it is of an acceptable scientific standard.

# Reviewer report: Reviewer response for version 2

**DOI:** 10.5256/f1000research.158405.r223167

**Published:** 2023-12-14

**Authors:** Muhammad Kashif Zahoor

**Affiliations:** 1 Department of Zoology, Government College University Faisalabad (GCUF), Faisalabad, Pakistan

The article has been improved. I don't have further comments.

Is the work clearly and accurately presented and does it cite the current literature?

Yes

If applicable, is the statistical analysis and its interpretation appropriate?

Partly

Are all the source data underlying the results available to ensure full reproducibility?

Partly

Is the study design appropriate and is the work technically sound?

Yes

Are the conclusions drawn adequately supported by the results?

No

Are sufficient details of methods and analysis provided to allow replication by others?

Yes

Reviewer Expertise:

Entomology, Genetics, Molecular Biology

I confirm that I have read this submission and believe that I have an appropriate level of expertise to confirm that it is of an acceptable scientific standard.

# Reviewer report: Reviewer response for version 1

**DOI:** 10.5256/f1000research.145570.r207554

**Published:** 2023-10-12

**Authors:** Muhammad Kashif Zahoor

**Affiliations:** 1 Department of Zoology, Government College University Faisalabad (GCUF), Faisalabad, Pakistan

The house fly has been considered a vector of more than a hundred diseases; which signifies its importance. It acts both as a biological as well as a mechanical vector. Different control measures have been devised but still this menace is not properly managed yet. The article titled 'Control of *Musca domestica *(Diptera, Muscidae) in Misan Province' is a good approach towards house fly control. 

 The manuscript, overall, is well written. However, minor changes are required in the Abstract, Introduction, Materials & Methods; whereas, major changes are required in the Results and Discussion sections before its indexing in its final version. 

 Title: Control study of *Musca domestica L. *(Muscidae: Diptera) in Misan Province

**Abstract section:**

**Methods: **

- "The study was conducted over 12 months...".
- Use *M. domestica *or house fly; don't use *Musca *flies in the manuscript.

**Keywords: **

- Italicize *Musca domestica*
- House fly (House flies are written with space not as Housefly).

**Introduction: **

- The very first word ... *Musca domestica*
- Different control measures (chemical control and environmental etc.) have been mentioned in this section. You should add some of the drawbacks/loopholes of these measures in favor of or to justify your methods applied in the present study. 

**Materials & Methods: **

- How were traps and papers placed/installed? Method of installation? How many traps/papers per house? 
- What did you put/insert inside the trap? Any chemical or pheromone? 
- If the larvae were identified? How?
- Green bottle/blue bottle flies also visit along with house flies; how can you differentiate them? Did you let them pupate and then to emerge as the adults for identification? It means you took alive larvae from the collection sites? 

 

**Figure 1: **

- Heading: Container for collecting house flies.

**Results:**

 First paragraph needs to be rephrased (in passive voice).

 The house selectionwas made randomly, ethical procedure was followed as prescribed by the institution. Out of 200 selected houses, 150 (75%) contained flies, while 50 (25%) did not have any larvae. The collection was made using traps and sticky papers. It is to mention that the houses were selected for being nearby large water sources and farms or trees, or regions of good conditions for grow-up of houseflies like moderate humidity and medium temperatures. The distribution of house flies in these habitats was as follows: indoors (48, 6.9%), outdoors (115, 16.8%), plastic cups (69, 10.0%), traps (184, 26.7%), oil papers (179, 25.9%), and sewage water (94, 13.7%) with a high statistical significance (P = 0.001). Light traps and sticky oil papers were the most effective control measures, with 26.7% and 25.9% of the house flies collected using these methods, respectively ( Table 2).

**Figure 2: **

- Head of male (A) and female (B) *Musca domestica* L. 
- Caption or legend needed for this Figure 1 which can describe what is A and B. Also you can indicate the difference between (A) and (B) with the help of arrows. 

**Table 2:** There is confusion in statistical analysis/values. Your sample size is 6 or 7 for number of houses, how is the df 4?

 Similarly for the number of *Musca domestica *your sample size is 6, so how df = 6 is possible? 

**Tables 3, 4 **- Your sample size is 2 in **Table 3** (male and female category) and **Table 4** (Hot months and cold month) while the author gave df = 2 in both Tables - how?  

 The **df **values throughout the article indicate that the author need to confirm the sample size/data for statistical analysis. Then also confirm if the subsequent analysis and other value i.e. P-values and X ^2^ be correct.

 The correct statistical analysis is a key to conclude the study in a pragmatic way; otherwise the results couldn’t be the actual presentation of what the study was aimed. I suggest you to correct the statistical analysis. 

**Discussion:**

 This section looks like the introduction section in the start while in the remaining part of the discussion, the results of the present study are poorly discussed with previous studies. Latest and recent references should be cited. Overall, this section needs to be improved. 

**Conclusions;** the author concluded that hot climate, humid sites and dirty places are responsible for the breeding of houseflies. Did the author evaluate these parameters in the present study? Where is the data representing these parameters?

 You should correlate your collected population of *Musca* domestica with these parameters. And if it’s reported in previous studies you can discuss in the discussion part instead of making it the conclusion of your present study.

Is the work clearly and accurately presented and does it cite the current literature?

Yes

If applicable, is the statistical analysis and its interpretation appropriate?

Partly

Are all the source data underlying the results available to ensure full reproducibility?

Partly

Is the study design appropriate and is the work technically sound?

Yes

Are the conclusions drawn adequately supported by the results?

No

Are sufficient details of methods and analysis provided to allow replication by others?

Yes

Reviewer Expertise:

Entomology, Genetics, Molecular Biology

I confirm that I have read this submission and believe that I have an appropriate level of expertise to confirm that it is of an acceptable scientific standard, however I have significant reservations, as outlined above.

# Response

**Published:** 2023-10-17

**Authors:** rasha alsaad

**Affiliations:** Faculty of medicine, Misan, Iraq

Hello Professor, thank you very much for the beautiful comments. I have completed the amendments that you suggested:

 Regarding the question about working methods: The traps were light-electric, working on light energy to catch all the insects, and then I would sort them according to their anatomical and morphological characteristics.

 As for the number of stickers, 2-3 are placed for each house in different places, in addition to one number of traps.

 F2 The difference between females and males and the method of separating them through anatomical and formal characteristics was explained.

 As for statistics, the statistician is the one who analyzed the samples.

 As for the conclusions, we have complete statistics about the climate of Maysan Governorate

 Thank you. I hope that my information is acceptable to you. Please accept my regards.

 Greeting with appreciation

# Reviewer report: Reviewer response for version 1

**DOI:** 10.5256/f1000research.145570.r201470

**Published:** 2023-09-19

**Authors:** Meltem Kökdener

**Affiliations:** 1 Department of Social Services, Faculty of Health Science, Ondokuz Mayis University, Samsun, Samsun, Turkey

I have made the necessary corrections to the text - please see the PDF file linked here for additional comments.

 The discussion part of the article can be expanded a little more, a map showing the study areas can be added, and the author can explain the work they plan to do in the future based on this study.

 The author should include the hypothesis of the study. It can be indexed after corrections and necessary additions are made. Make sure that all references are included in the text.

Is the work clearly and accurately presented and does it cite the current literature?

Yes

If applicable, is the statistical analysis and its interpretation appropriate?

Yes

Are all the source data underlying the results available to ensure full reproducibility?

Yes

Is the study design appropriate and is the work technically sound?

Yes

Are the conclusions drawn adequately supported by the results?

Yes

Are sufficient details of methods and analysis provided to allow replication by others?

Yes

Reviewer Expertise:

entomology, forensic entomology

I confirm that I have read this submission and believe that I have an appropriate level of expertise to confirm that it is of an acceptable scientific standard.

# Response

**Published:** 2023-10-11

**Authors:** rasha alsaad

**Affiliations:** Faculty of medicine, Misan, Iraq

I am sending you my research after making the modifications that suit the research's goal, science, and purpose. Some were neglected because they were mentioned in the research but in another place. Please accept my regards

 Rasha
